# Report of the Physicians Who Attended President McKinley

**Published:** 1901-11

**Authors:** 


A Monthly Review of Medicine and Surgery.

EDITOR:

WILLIAM WARREN POTTER, M. D.

All communications, whether of a literary or business nature, books for review and exchanges should be addressed to the editor: 284 FRANKLIN STREET, BUFFALO, N. Y.

VOL. XLI.—LVII. NOVEMBER, 1901. NO. 4.

### Report of the Physicians who Attended President McKinley.

THE official report of the President's medical and surgical staff, printed elsewhere, presents many points of interest, though most of the facts it contains already have been published in the JOURNAL. One of the striking features of the case which its reading accentuates was the low vitality of the President,—a factor of great import in determining the fatal issue.

President McKinley had been in public life for 40 years, the last decade of which it will be remembered was a period of continual strain. While he was in comparative health when stricken down, and was able to meet the every-day demands upon his physical resources, yet he was unprepared to withstand the enormous drain and strain that the last seven days of his life required. It early became necessary to resort to cardiac stimulants and these were but poorly or at most only temporarily responded to. The reparative force was not there in sufficient volume to meet the requirements of such a sudden and awful exaction. The solar plexus undoubtedly received a shock both from the wound and the necessary surgical manipulations that contributed to the complications of the case.

The autopsy report in all its details is of special interest and throws much light upon points heretofore not clearly comprehended by the profession or the public. The bacteriological investigation was most complete and the report of it contributes to the general fund of information in a very satisfactory manner. The minute and painstaking character of the two latter—anatomical and bacteriological—will attract attention, and the entire report in its several parts will convince the world, lay as well as professional, that the President of the United States in his grievous affliction received the very best medical and surgical care, that nothing was left undone which could have contributed to his comfort or safety, and that his wound was one in its very nature which no skill in surgery could cure, it being absolutely mortal in its effects, and beyond all but temporary relief.

The entire conduct of the case was upon modern scientific medical and surgical principles, and no intelligent observer can gainsay this fact, which is a comforting reflection in view of the awfulness of a calamity that plunged the whole world into the deepest gloom. These are some of the reflections we are led into after reading this report, which is a becoming final chapter to a history that saddens the hearts of our people more universally than any other event since the foundation of the republic.

